# Machine-learning-derived classifier predicts absence of persistent pain after breast cancer surgery with high accuracy

**DOI:** 10.1007/s10549-018-4841-8

**Published:** 2018-06-06

**Authors:** Jörn Lötsch, Reetta Sipilä, Tiina Tasmuth, Dario Kringel, Ann-Mari Estlander, Tuomo Meretoja, Eija Kalso, Alfred Ultsch

**Affiliations:** 1 Institute of Clinical Pharmacology, Goethe - University, Theodor – Stern - Kai 7, 60590 Frankfurt am Main, Germany; 2 Fraunhofer Institute for Molecular Biology and Applied Ecology IME, Project Group Translational Medicine and Pharmacology TMP, Theodor – Stern - Kai 7, 60596 Frankfurt am Main, Germany; 3 Pain Clinic, Department of Anaesthesiology, Intensive Care and Pain Medicine, Helsinki University Hospital and University of Helsinki, Helsinki, Finland; 4 Breast Surgery Unit, Comprehensive Cancer Center, Helsinki University Hospital and University of Helsinki, Helsinki, Finland; 5 DataBionics Research Group, University of Marburg, Hans – Meerwein - Straße, 35032 Marburg, Germany

**Keywords:** Pain, Bioinformatics, Data science, Chronification

## Abstract

**Background:**

Prevention of persistent pain following breast cancer surgery, via early identification of patients at high risk, is a clinical need. Supervised machine-learning was used to identify parameters that predict persistence of significant pain.

**Methods:**

Over 500 demographic, clinical and psychological parameters were acquired up to 6 months after surgery from 1,000 women (aged 28–75 years) who were treated for breast cancer. Pain was assessed using an 11-point numerical rating scale before surgery and at months 1, 6, 12, 24, and 36. The ratings at months 12, 24, and 36 were used to allocate patents to either “persisting pain” or “non-persisting pain” groups. Unsupervised machine learning was applied to map the parameters to these diagnoses.

**Results:**

A symbolic rule-based classifier tool was created that comprised 21 single or aggregated parameters, including demographic features, psychological and pain-related parameters, forming a questionnaire with “yes/no” items (decision rules). If at least 10 of the 21 rules applied, persisting pain was predicted at a cross-validated accuracy of 86% and a negative predictive value of approximately 95%.

**Conclusions:**

The present machine-learned analysis showed that, even with a large set of parameters acquired from a large cohort, early identification of these patients is only partly successful. This indicates that more parameters are needed for accurate prediction of persisting pain. However, with the current parameters it is possible, with a certainty of almost 95%, to exclude the possibility of persistent pain developing in a woman being treated for breast cancer.

**Electronic supplementary material:**

The online version of this article (10.1007/s10549-018-4841-8) contains supplementary material, which is available to authorized users.

## Introduction

Breast cancer is the most common cancer in women in the developed countries and its prevalence is steadily increasing [3, 15, 17]. Improved management of breast cancer has made survivorship issues important [41], including moderate to severe pain reported with a prevalence of about 15% at 1 year from surgery [37]. About 34% of these patients have signs of neuropathic pain [48] and the pain can last for several years [36], significantly impairing their quality of life [52]. Prevention of persistent post-surgical pain requires early identification of patients at the highest risk to initiate appropriate medical and psychosocial interventions [10, 43]. On the other hand, identifying patients in whom the possibility of persistent pain can be dismissed with high confidence may be similarly desirable as this could prevent unnecessary therapeutic interventions.

The prediction of persistent pain after a surgical intervention is therefore an active research topic that has already led to several proposed solutions, as indicated by 160 hits in a PubMed search at https://www.ncbi.nlm.nih.gov/pubmed for “(chronic or persistent) and pain and prediction and *surgery” on December 6, 2017. However, diagnostic tools providing such a prediction have remained an unmet clinical need. Considering that pain has a highly complex pathophysiology [22, 47] and is triggered by several different causes including cancer [44] and surgery [25], such tools may require a combination of different parameters. However, in addition to proposals of biochemical or genetic markers, clinical and psychological parameters have remained a basis of predictive assessments of the development of pain after surgery [4, 11, 30, 37].

In the present analysis, more than 500 parameters were available from a 3-year follow-up of 1,000 women operated on for breast cancer [49]. This provided a robust basis for clinical judgment of pain persistence and, using parameters acquired during the perioperative period, allowed the prediction of either persistent pain at later stages or its unlikeliness. Previous analyses indicated the suitability of these parameters for the prediction of persistent pain [32, 49]; however, the present analysis used a data-driven approach, without prior hypotheses on the most important parameters, to create a predictive diagnostic tool for persistent pain, or its absence, following breast cancer surgery and adjuvant therapies.

## Methods

### Patients

**Fig. 1:**
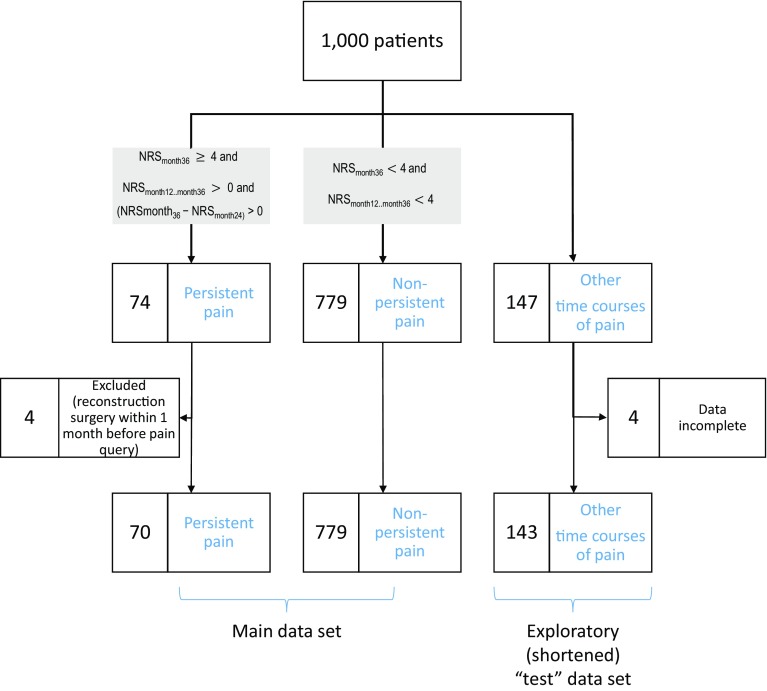
Flow chart showing the classification of the patients on the basis of the 3-year development of pain following breast cancer surgery. A total of 853 women fell into the two main groups of persisting or non-persisting pain, according to the criteria displayed in the gray-shaded frames. This was the main cohort that was analyzed. The remaining 143 women in whom the criteria for class assignment applied only partly were therefore excluded from machine-learned classifier establishment but they were used as an exploratory shortened “test” data set. Incomplete returns of pain questionnaires were dealt with by imputation as detailed in the methods section

The study followed the Declaration of Helsinki, and both the Coordinating Ethics Committee (journal number 136/E6/2006) and the Ethics Committee of the Department of Surgery (148/E6/05) of the Hospital District of Helsinki and Uusimaa approved the study protocol. Written informed consent for data acquisition and publication in an anonymized manner was obtained from each participating patient. One thousand women with unilateral non-metastasized breast cancer, aged 28–75 years (Fig. 1) were enrolled during the preoperative visit before breast cancer surgery. They were treated at the Helsinki University Hospital between 2006 and 2010 with breast-conserving surgery or mastectomy, and sentinel node biopsy and/or axillary clearance. Exclusion criteria were neoadjuvant therapy, i.e., administration of chemotherapy to shrink the tumor before the main surgical treatment [54], and immediate breast reconstruction surgery. Of the 1,536 consecutive eligible patients, 1,149 patients were invited to participate, of whom 126 patients declined and 23 were withdrawn.

The cohort has previously been described in detail [24, 37]. Briefly, perioperative analgesia was standardized, consisting of oral acetaminophen and intravenous oxycodone, titrated first by a research nurse in the post-anesthesia care unit followed by patient-controlled analgesia on the ward. No regional anesthesia was used. Analgesia at home during the first postoperative week consisted of ibuprofen, acetaminophen or a combination of acetaminophen and codeine. Adjuvant treatments were given according to international guidelines [37].

### Data acquisition

#### Acquisition of data on pain during the follow-up

A full listing of the acquired data is provided in Supplementary Table 1. Pain was assessed using a standard 11-point Numerical Rating Scale (NRS) ranging from 0 to 10, 0 indicating “no pain” and 10 the “worst imaginable pain” [12]. The mean daily post-surgical pain intensity was recorded during the first postoperative week with patient-rated paper diaries. At 1, 6, 12, 24, and 36 months after surgery, questionnaires were posted to all patients asking about the presence, location, and intensity of pain, pain interference, and mood. The patients reported, as a single rating, the worst pain, either at rest or during movement, in any of the surgery-related locations (breast, axilla, upper arm) during the previous week. The questionnaires about pain interference with daily life and sleep were developed for this study. The presence or absence of persistent pain was established from these questions at 12–36 months.

#### Acquisition of candidate parameters for prediction of pain persistence

To predict the development or absence of persistent pain after breast cancer surgery, the so-called “input space” of 542 different variables was acquired from the data collected before surgery, during the perioperative phase, and at follow-up until 6 months after surgery. The acquired variables, in the present context of machine-learning [39] called parameters or “features,” included the patients’ medical history (diseases, medications, number of previous surgeries), and demographic data. In addition, pain-related data included the presence of persistent pain of any kind, preoperative pain in the operated area (breast, axilla, arm), opioid (remifentanil) consumption during surgery, immediate postoperative pain intensity ratings (at rest and during movement), amount of oxycodone needed for satisfactory pain relief for the first time after surgery, oxycodone consumption during 20 h after surgery, pain intensity, and analgesic consumption during the first postoperative week. The parameters also included surgical data such as type of surgery (mastectomy or breast-conserving surgery, axillary clearance, sentinel node biopsy), complications of surgery and re-operations, pathology data such as tumor and lymph node characteristics. Finally, data on adjuvant therapies (chemotherapy, radiation therapy, endocrine therapy) were acquired.

In the follow-up questionnaires, detailed pain-related parameters about specific pain locations were sought and the patients were asked whether the pain had disturbed their sleep or otherwise affected their life (11-points NRS ranging from 0 to 10, 0 indicating “not at all” and 10 “very much”), and what analgesics they used, if any. Psychological data were acquired with questionnaires including Beck’s Depression Inventory (BDI) [2] and Spielberger’s State-Trait Anxiety Inventory (STAI) [51] before surgery and at the follow-up times. The State-Trait Anger Expression Inventory (STAXI2) [50] was used preoperatively and at 6 months. All data were entered manually into Excel files and double-checked by two investigators.

### Data analysis

Data analysis was performed using the MATLAB numerical computing environment (version 8.3.0.532, MathWorks, Natick, MS, USA) and R software package (version 3.2.3 for Linux; http://CRAN.R-project.org/ [45]).

Application of the previously proposed six-factor risk index for persisting pain was performed according to the instructions in Table A1 in the appendix of [49]. In brief, the six items “age,” “chronic pain of any kind,” “number of previous operations,” “body mass index,” “preoperative pain in the area to be operated on,” and “smoking” were weighted according to these instructions. For example, age ≤ 39 years was given a weight of 0, age 40–69 years a weight of 8, and age ≥ 70 years a weight of 16. The respective weights for the six parameters were added from an index of 20, patients were predicted as potentially developing persisting pain.

**Fig. 2:**
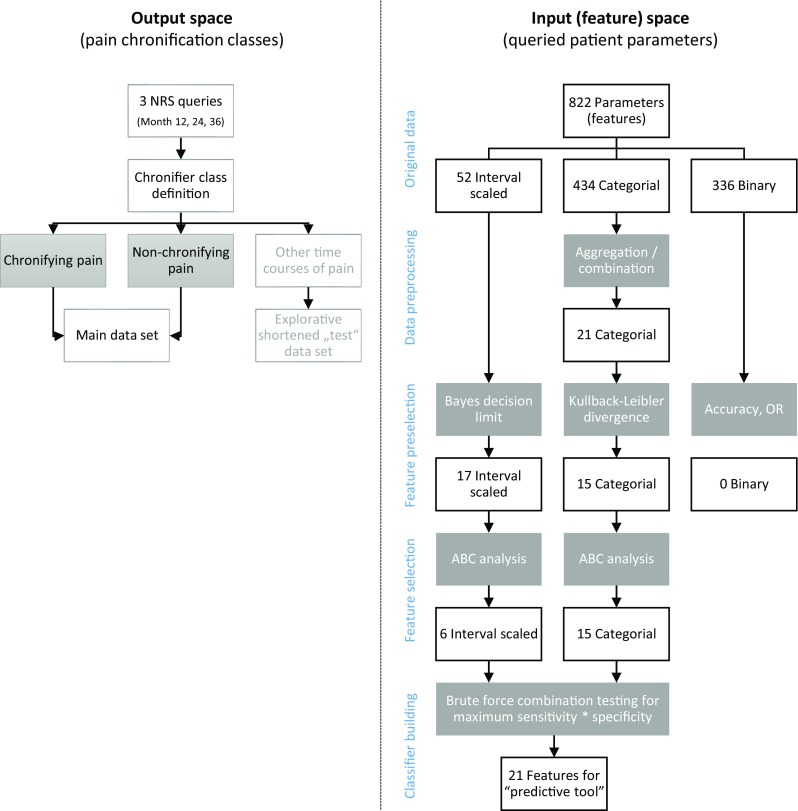
Flow chart of the data analysis. The figure provides an overview on the applied machine-learning approach in four steps (indicated in blue: output space preparation, input space feature pre-selection, feature selection and classifier building, including validation). The white frames show the variable flow; the gray frames depict the bioinformatics operation applied on the variables. During feature pre-selection and feature selection, the number of candidate variables qualifying as component s of a diagnostic tool respectively classifier was stepwise reduced (initially 542, finally 21), forwarding to the next analytical step only those features that had passed the criteria of the actual selection procedure. The Bayesian decision limit and Kullback–Leibler divergence refer to the respective standard procedure presented elsewhere [28, 35]

Given the broader data basis available in the present analysis, a novel and independently designed analysis was performed to assess whether the classification performance could be improved. This was approached using supervised machine-learning [39] and feature-selection techniques [19], aimed at identifying parameters from the data acquired before surgery and up to the sixth month after surgery, which could predict the presence or absence of persisting pain in the area operated on during the 3-year follow-up available for the present analysis. However, as before [49] an interpretable and clinically immediately applicable diagnostic tool was desired and therefore, a rule-based classifier [59] was again chosen forming a questionnaire with “yes/no” items (decision rules). The analysis was performed in four main steps as shown in Fig. 2 and comprising (i) establishment of the so-called output space, (i) feature pre-selection, (iii) feature selection, and (iv) classifier building, which are described briefly in the following; more detailed descriptions are provided in the Supplementary materials.

**First**, the so-called “output space” was established (Fig. 2 left part) by identifying the groups or “classes” of patients with respect to persistent pain. In the following, the term “persisting pain group” and “non-persisting pain group” or “classes” will be used when referring to these groups. Subsequently, the input or feature space was analyzed to identify those parameters that provided a valid assignment of a patient to the classes of persisting or non-persisting pain. This included the **second** analytical (Fig. 2 right part) step comprising a “feature” pre-selection, during which all parameters were analyzed with respect to differences between the “persisting pain” and “non-persisting pain” groups, established in the output space. This was based on the assessment of effect sizes between the two patient groups depending on the numerical scaling of the parameters as continuous (interval-scaled), discrete or binary (“yes”/”no”).

Those parameters that had passed that step were taken into the **third** analytical step of feature selection (Fig. 2 right part), which eliminated all parameters distinguishing between the persisting pain and non-persisting pain groups but not providing additional relevant information justifying their inclusion in a tool (“classifier”) predictive of persisting pain. This applied a so-called calculated ABC analysis [56]. This is an inventory categorization technique originally developed for problems in economics to search for a subset with the minimum possible effort that gives the maximum yield [23, 42]. The remaining parameters, in the following called “features,” were taken into the **fourth** step of the data analysis (Fig. 2 bottom right) in which the predictive tool or “classifier” was constructed and assessed with respect to standard test performance measures. To obtain a robust selection of parameters (features), Monte-Carlo resampling was used repeatedly.

## Results

### Persisting versus non-persisting pain groups

The recovery rate of the pain questionnaires was high with 95.3, 91.3, 90.2, 90.2, and 87.4% returned in months 1, 6, 12, 24, and 36, respectively. This sufficed for a valid classification of the subjects into two main diagnostic groups that displayed temporal courses of pain for 3 years after breast cancer surgery obtained in the first analytical step aimed at establishing the so-called output step (Fig. 2 left). Of the 1000 analyzed women (Fig. 1), 779 had a favorable time course of postoperative pain and belonged to the “non-persisting pain” group characterized by NRS_month12...month36_ ≤ 3 (Fig. 1 left). By contrast, in 74 women, the pain followed the opposite path typical for the “persisting pain” group characterized by NRS_month36_ ≥ 4 and NRS_month12...month36_ > 0 and (NRS_month36_ − NRS_month24_) ≥ 0 (Fig. 1 middle). Four of these women were excluded from the analysis due to breast reconstruction surgery within the previous month, which hampered the clear association of pain at month 36 to the original surgery. Finally, the criteria for class assignment were not completely met in the remaining 143 women who were therefore excluded from machine-learned classifier establishment (Fig. 1 right) but were used as an exploratory “test” data set.

### Classifier establishment

**Fig. 3:**
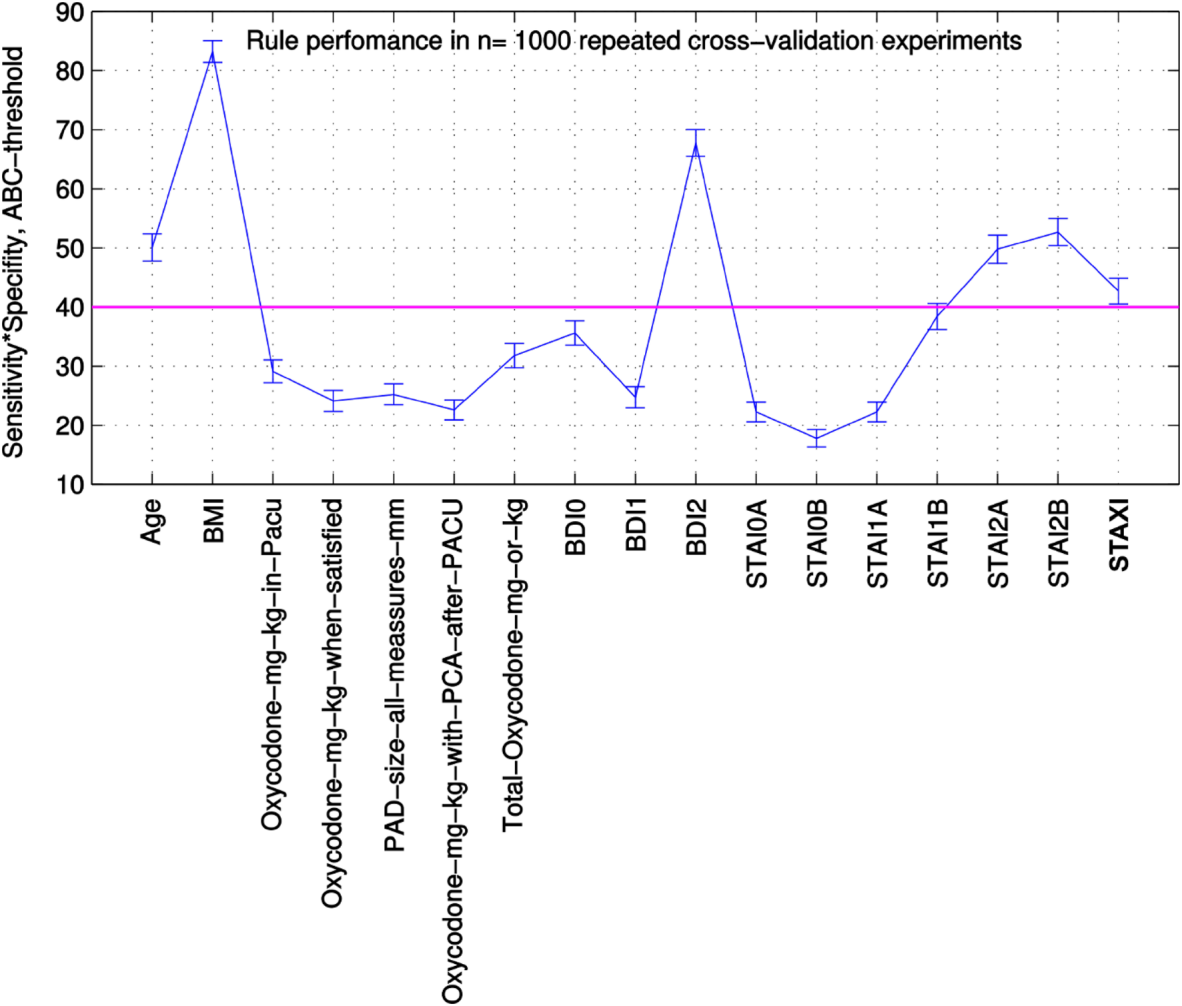
Performance of the continuous variables with a Bayesian decision boundary in 1,000 repeated cross-validations. The *n* = 17 continuous variables were subjected to an ABC analysis (for ABC analysis, see [56]). The set A (best performers) was characterized by a sensitivity · specificity > 40% (threshold; magenta line). The resulting 6 variables in set A were included in the classifier construction. Names of variables above the threshold: Age, BMI, BDI0 = preoperative BDI, BDI1 = BDI at 1 month after surgery, BDI2 = BDI at 6 months after surgery, STAI0A, STAI1A, STAI2A = State anxiety (STAI) aquired preoperatively and at 1 month and 6 months after surgery, respectively, STAI0B, STAI1B, STAI2B = Trait anxiety (STAI) aquired preoperatively and at 1 and 6 months postoperatively, respectively, STAXI = Anger inhibition (STAXI)

Supervised machine-learning was applied to map the “input space,” i.e., the acquired “features” (the 542 parameters), *x*, to the “classes,” i.e., the outputs, *y*, given the data subset of the input–output pairs $D=\left\{ {\left( {{x_i},{y_i}} \right)|{x_i} \in X,~{y_i} \in Y,~i=1 \ldots n} \right\}$ that comprised the *n* = 849 patients belonging to the two groups (persisting or non-persisting pain). Feature pre-selection (data analysis step 2; Fig. 2 right part) identified 39 parameters for which the analysis of probability of belonging to the “persisting pain” group, i.e., $p\left( {y={y_1}|x,D} \right)$, was in principle possible. During feature selection (data analysis step 3; Fig. 2 right part), among these 39 candidates, ABC analysis picked 21 parameters (features) six continuous and 15 discrete variables (Fig. 2) that provided a substantial contribution to the sensitivity and specificity of a classifier for a patient’s assignment to one of the groups (persisting pain or non-persisting pain). The process of feature selection via 1,000 resampling and ABC analyses is shown in Fig. 3 for the 17 continuous variables, of which only six passed the analytical step due to their consistent placement in set “A” comprising the parameters most suitable as predictors.

**Fig. 4:**
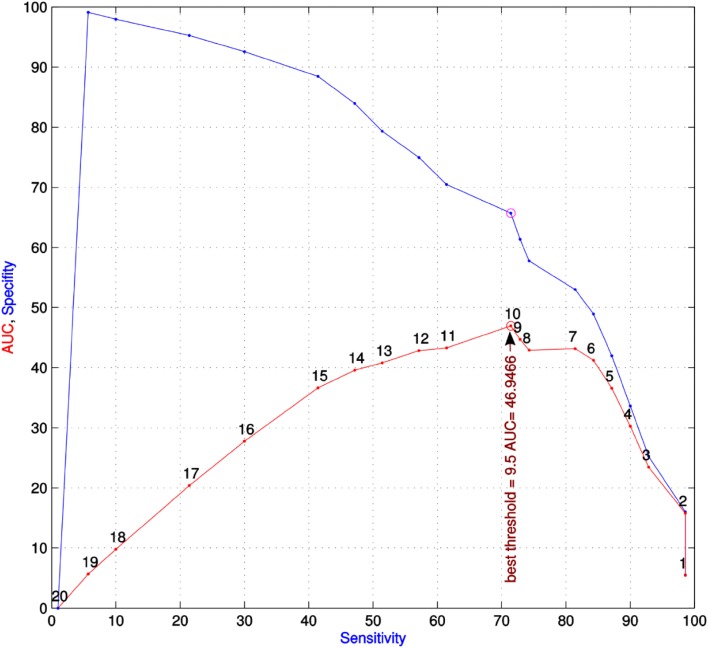
Plot of the specificity versus the sensitivity of using all possible combinations and thresholds for the 21 candidate predictors of persistent pain after breast cancer surgery (classifier construction). The number of conditions for a positive classification into the “persisting pain” groups ranges from *n* = 1–20 conditions. For all of these positive conditions, the sensitivity, specificity, and the area under the curve (AUC = sensitivity · specificity) was calculated. The red dots in the figure show AUC versus sensitivity. The black numbers close to the red dots indicate the number of conditions to be true according to the questions in Table 2. The maximum AUC, i.e., the best number of conditions for a classifier, was obtained with at least 10 positive items from Table 2, which was the result of the analysis shown in this figure and the reason why the final predictive tool required 10 or more positive items. The blue dots in the blue line indicate the corresponding specificity (ordinate) versus sensitivity (abscissa) values. The lines have been drawn to enhance visibility and are spline interpolations

A rule-based classifier [59] was obtained during analytical step 4 (Fig. 2 bottom right). It took the shape of a diagnostic tool shown in Table 2 consisting of a set of items presented as “yes” or “no” decisions. The diagnostic tool comprised three main categories of variables, i.e., demographic, psychological, and pain-related parameters (features). For each item, the prediction of persistent pain, i.e., the assignment of a patient to the “persisting pain” group (output space), was likely at a specific threshold shown in the right column of Table 2. The number of positive decisions by which a patient was classified into the “persisting pain” group was established testing all possible feature combinations. This analysis resulted in a threshold of 9.5 (Fig. 4), which indicates that when at least 10 of the 21 rules listed in Table 2 applied the patient was identified as likely to develop persistent pain. The detailed use of the diagnostic tool is described step-by-step in Textbox 1.

**Table 1:** Parameter list including original features along with the details of feature aggregation

Number	Parameters (features)	Time				Body location							
		Preoperative	Week 1	Week 4	Week 24	Breast	Axilla	Upper arm	Joints	Lower arm	Hand/fingers	Operated side arm	Somewhere
1	Age	X											
2	BMI	X											
3	Depressive symptoms (BDI)				X								
4	State anxiety (STAI)				X								
5	Trait anxiety (STAI)				X								
6	Anger inhibition (STAXI-2)	X											
7	Have the pains in the extremities* affected your life?			X			X	X	X	X	X		
8	Have the pains in the axilla affected your life?		X	X	X		X						
9	Have the pains in the hand or fingers affected your life?		X	X	X						X		
10	Have the pains in the joints affected your life?		X	X	X				X				
11	Have the pains in the lower arm affected your life?		X	X	X					X			
12	Have the pains in the upper arm affected your life?		X	X	X			X					
13	How much has the pain disturbed your sleep?			X									
14	How much has the pain disturbed your sleep?				X								
15	How much has the pain in the axilla disturbed your sleep?			X	X		X						
16	How much has the pain in the breast disturbed your sleep?			X	X	X							
17	Pain intensity in the operated-side arm and axilla in the morning?		X	X			X					X	
18	Worst pain intensity during the past week at one month			X		X	X	X	X	X	X		
19	Worst pain intensity during the past week at 6 months				X	X	X	X	X	X	X		
20	Worst pain intensity during the past week in the operated breast?			X	X	X							
21	Worst pain intensity during the past week somewhere?			X	X								X

The table specifies the time points and, if applicable, the targeted body locations for several questions the patients are asked following surgery. Specifically, for the pain-related parameters #7 - #21 (according to the numbering in the left column), patients were asked about how the pain affected their lives or sleep. These questions were asked at several times (week 1, 4, and 24 after surgery) and for several specific body locations (breast, axilla, upper arm, joints, lower arm, hand, arm on operation side, elsewhere) indicated in the middle block of the table. The “X”s indicate which ratings were averaged to obtain the parameters

*Extremities: aggregated parameters across axilla, upper arm, lower arm, hand/fingers, and the associated joints

**Table 2:** Parameters (predictive factors) for persisting pain following breast cancer surgery

Number	Category	Parameters (predictive factors)	Threshold
1	Demographic factors	Age	> 62
2		BMI	> 31.5
3	Psychological factors	Depressive symptoms (BDI)	> 11
4		State anxiety (STAI)	> 35
5		Trait anxiety (STAI)	> 37
6		Anger inhibition (STAXI)	> 12
7	Pain-related factors	Have the pains in the extremities *affected your life?	> 1.5
8		Have the pains in the axilla affected your life?	> 0.5
9		Have the pains in the hand or fingers affected your life?	> 0.5
10		Have the pains in the joints affected your life?	> 0.5
11		Have the pains in the lower arm affected your life?	> 0.5
12		Have the pains in the upper arm affected your life?	> 0.5
13		How much has the pain disturbed your sleep?	> 0.5
14		How much has the pain disturbed your sleep?	> 0.5
15		How much has the pain in the axilla disturbed your sleep?	> 0.5
16		How much has the pain in the breast disturbed your sleep?	> 0.5
17		Pain intensity in the operated-side arm and axilla in the morning?	> 2.5
18		Worst pain intensity during the past week at one month	> 1.5
19		Worst pain intensity during the past week at 6 months	> 0.5
20		Worst pain intensity during the past week in the operated breast?	> 1.5
21		Worst pain intensity during the past week somewhere?	> 1.5
All		“Persistent pain” class if sum of positively answered items ≥ 10	

A patient is likely to develop persistent pain if at least 10 of the 21 items (rules) apply

*BMI* body mass index, *BDI* Beck’s Depression Inventory, *STAI* Spielberger’s State-Trait Anxiety Inventory, *STAXI* Spielberger’s State-Trait Anger Expression Inventory, *m* months

### Textbox 1: Summary of the data acquisition and calculation steps required to apply the diagnostic tool, as presented in Table 2, to a single patient. See Table 1 for details about the items that need to be averaged

- Observation period for application of the predictive tool defined in Table 2 is 6 months after breast cancer surgery.
- Query of demographic factors (age, BMI; items 1 and 2 in Table 1).
- Query of complex psychological questionnaires (STAXI, preoperative), BDI, STAI state, and STAI trait at week 24 postoperative (**items 3–6 in** Table 1).
- Query of pain-related ratings addressing how much the pain has affected the patient’s life or sleep during the last week, or querying the pain intensity, globally and also separately for various different body locations (items 7–21 in Table 1).
  - How much pain has affected the patient’s life is queried using an 11-point NRS ranging from 0, not at all, to 10, very much (items 7–12 in Table 1). Items are queried at week 1, 4, and 24 at various body locations and averaged for week 4 across all body locations and for each of six body locations (breast, axilla, upper arm, joints, lower arm, hand/fingers) averaged across weeks 1, 4 and 24.
  - How much pain has affected the patients sleep is queried using an 11-point NRS ranging from 0, not at all, to 10, very much (items 13–16 in Table 1). Items are queried at week 4 and 24, globally (**items** 13 and 14 in Table 1), or asked specifically for the effect of pain at body locations breast or axilla. Items are queried at week 4 and 24 for the two body locations are averaged per body location (items 15 and 16 in Table 1).
  - Pain intensity in the operated-side arm and axilla (NRS from 0, no pain, to 10, worst imaginable pain), in the morning is queried at week 4 and 24 and averaged across the time points (item 17 in Table 1)
  - The worst pain intensity experienced during the past week (items 18–21 in Table 1) is queried for six body locations (breast, axilla, upper arm, joints, lower arm, hand/fingers) and in addition, for any part of the body (“somewhere”) using an 11-point NRS ranging from 0, not at all, to 10, very much. Items are queried at week 4 and 24 averaged per week across all body locations (**items 18 and 19 in Table 1**). Ratings for pain in the operated breast or “somewhere” are averaged across weeks 4 and 24 (items 20 and 21 in Table 1)
- Each of the 21 items (Table 1) obtained as described above is assessed with respect to the Bayesian threshold (Table 2, right column) and the sum of the “yes”/”no” answers to the question: “Above threshold?” are added.
- Persistent pain is likely when the above sum equals 10 or higher.

### Classifier performance

At the end of analytical step 4, the performance of the obtained classifiers to correctly assign a patient to the persisting pain groups was tested and compared with that of the previously proposed six-factor risk index for persisting pain [49] comprising the weighted items “age,” “chronic pain of any kind,” “number of previous operations,” “body mass index,” “preoperative pain in the area to be operated,” and “smoking,” This provided a performance of 70.6% sensitivity and 45.4% specificity for correct assignment of a patient to the persisting pain group. The accuracy for correct group prediction was 47.4%, and the balanced accuracy, which takes unequal group sizes into account, was 57.9%. However, the negative predictive value, which quantifies the probability that persisting pain will not develop when the test is negative, was high with 94.5% correctly excluding a development toward persistent pain.

The novel, more complex, diagnostic tool provided, via 1000 Monte-Carlo random resamplings [18] of 50% of the original data set, a cross-validated classifier performance of 79.3 ± 1.5% sensitivity and 51.4 ± 6% specificity for correct assignment of a patient to the persisting pain group. The overall classification accuracy obtained in the 1,000 runs with resampled data was 86.2 ± 1.4%, and the balanced accuracy was 65.4%. The negative predictive value was again high with 94.8% (binomial confidence interval: 93.4–95.9%).

Finally, the classifier performances were assessed on the 143 patients who did not completely meet the strict criteria of persistent pain but nevertheless displayed an unsatisfactory course of pain development and were therefore used as an exploratory “test” data set. Thus, when only the first criterion of the NRS-based classification into persisting and non-persisting pain groups was applied, i.e., patients with NRS ≤ 3 at month 36 after surgery (*NRS*_month36_ ≤ 3) were identified as belonging to the “non-persisting pain” group, while those with (NRS_month36_ ≥ 4) had “persisting pain,” 143 patients could be classified. Of these, 21 patients had at 3 years a pain score of ≥ 4 and were therefore considered as having an unfavorable clinical outcome. These patients were thus defined as “presumably having persisting pain” and therefore belonging to the “persisting pain” group. Taking the aforementioned unfavorable outcome as a sign of persisting pain, the 6-item classifier provided sensitivity and specificity of identifying potentially persisting pain of 94.1 and 43.7%, respectively, and an accuracy or balanced accuracy of class assignment of 46.6 or 66.9%, respectively. The more complex novel classifier provided 14 true positive, 3 false negative, 56 false positive, and 70 true negative diagnoses of persistent pain. This translates into sensitivity and specificity of identifying a patient at risk of 82.4 and 55.6%, respectively. The accuracy was 58.7%, the balanced accuracy was 69%, and a negative predictive value of 95.9% was obtained (binomial confidence interval: 88.5–99.1%).

## Discussion

The main result from the present machine-learned analysis was that a more complex diagnostic tool than previously proposed for a subgroup of 489 of the present patients [49] improved the correct assignment of a patient to either the persistent or non-persistent pain group, raising accuracy to 86% and balanced accuracy to 65.4%, while maintaining the already high negative predictive value for persistent pain of 95%.

The present criteria of persistent pain differed from the proposal of the International Association for the Study of Pain in two aspects, i.e., (i) the time window was longer, six months instead of two, due to the particular clinical setting of breast cancer surgery, and (ii) the presence of relevant preoperative pain jeopardizing the attribution of postoperative pain to the surgery. Eleven patients of the “persisting pain” group had an NRS value > 3 for preoperative pain. In nine of the 74 patients assigned to the “persisting pain” group (12%), pain at 36 months was less although still > 3/10 NRS. Thus, the definitive criteria for postsurgery pain were not met in these patients and the predictor correctly addressed persistent pain in a breast cancer setting without unequivocally implying a surgical procedure as a causal factor. This should be considered when interpreting and applying the present diagnostic tool.

While in the original 6-item index “age,” “chronic pain of any kind,” “number of previous operations,” “body mass index,” “preoperative pain in the area to be operated,” and “smoking” were included, the novel selected features differed partly from this earlier selection. This probably owes to the longer observation period of the present analysis, and to the different techniques of feature selection. Nevertheless, some parameters agreed including a first major category of predictive features comprised demographic parameters. An increasing prevalence of chronic pain with age has been shown in several epidemiological studies [14]. The prevalence of neuropathic pain is also significantly higher in the elderly [53]. Interestingly, many previous, mainly cross-sectional studies, have reported that younger age would increase the risk for persistent pain after breast cancer treatment [1]. Previous studies have usually included mild pain intensities, whereas we used at least moderate pain as a cut-off. Younger women may report more discomfort related to milder pain due to a more active life style. Similarly, high body weight and chronic pain have been suggested to represent significant comorbidities, adversely impacting each other [40]. The association is not specific to a particular kind of pain but has been seen across several different aetiologies and clinical conditions [8, 20, 38, 40, 46, 60]. Possible physiological moderators include low-grade systemic inflammation and metabolic disorders [7, 29].

The second major category of predictive features emphasized the importance of psychological factors for the persistence of pain. This is in line with evidence that psychological factors play an important role in pain persistence [4, 5, 11, 16, 27, 30, 31, 33, 34, 37]. Psychological factors and pain have bidirectional influences, i.e., psychological factors influence how the patient perceives and interprets pain and vice versa, constant pain may affect psychological factors and have an impact on mood either directly or via its negative effects on sleep, functionality, social, and other activities [57, 58]. Thus, psychological factors may be at the same time predictors, maintainers, changeable variables and consequences of persistent pain [13]. Therefore, psychological factors have been proposed as early predictive markers for the development of persisting pain (e.g., [37]).

In the present study, women at risk of developing persistent pain reported more symptoms of depression and state and trait anxiety. A higher tendency to inhibit anger, as assessed before surgery, was related to pain persistence. Anger inhibition has been suggested to be closely related to general negative affect and depressive symptoms [6], and thus to associate with chronic pain [21]. Similar associations of chronic pain with psychological factors have been reported in several clinical settings not restricted to cancer surgery [5, 16, 27, 31, 33, 34]. Feelings of anxiety and lowered mood preoperatively are natural reactions to a serious disease, especially immediately after diagnosis. Interestingly, the level of psychological wellbeing at 6 months predicted persistent pain at 3 years, suggesting that better psychological coping after the acute phase might associate with lower risk of pain persistence. It might therefore be important to include routine assessment of psychological coping at this time point and initiate appropriate interventions as needed.

Finally, the third major category of predictive features was related to pain. These features were present early on and continued to persist for up to 3 years. Indeed, this is a recognized observation and, due to this, early treatment of pain has become a clinical routine. Interestingly, the early perception that pain impacted the patient’s life did not closely correlate with pain intensity. This was indicated by the lack of significant correlation between the ratings of the magnitude of the impact on the life and the associated NRS ratings of the pain intensity at the first month after surgery (Kendall’s τ = 0.136) [26, 55].

## Conclusions

Using a data-driven approach in a cohort of 1,000 women operated on for breast cancer, a set of demographic, psychological, and pain-related parameters available not later than at 6 months after surgery was associated with the persistence of pain in a 3-year follow-up. Applying machine-learning techniques, a classifier was developed that finally took the shape of a questionnaire with “yes/no” decisions about clinical features. Its main clinical strength lies in the exclusion of the possibility of developing persistent pain in a woman being treated for breast cancer with an accuracy of 95% (negative predictive value). This provides a clinically reliable basis to release a patient early from multidisciplinary therapy approaches [9]. However, with not more than 80% sensitivity and roughly 70% specificity, the performance of the novel classifier in predicting chronic pain in a patient remained only moderate. This suggests that the demographic, psychological, and clinical parameters included in the present classifier are insufficient as predictors of persistent pain. Future improvements should be searched for in “omics,” i.e., features based on proteomics, lipidomics, or genomics, to achieve the clinically desired early identification of patients who will develop persistent pain following surgery for breast cancer.

## Electronic supplementary material

Below is the link to the electronic supplementary material.

Supplementary material 1 (DOCX 43 KB)

Supplementary material 2 (XLSX 19 KB)

## References

[1] Andersen KG, Kehlet H (2011). Persistent pain after breast cancer treatment: a critical review of risk factors and strategies for prevention. J Pain.

[2] Beck AT, Ward CM, Mendelson M, Mock JE, Erbaugh JK (1961). An inventory for measuring depression. Arch Gen Psychiat.

[3] Bray F, Ren JS, Masuyer E, Ferlay J (2013). Global estimates of cancer prevalence for 27 sites in the adult population in 2008. Int J Cancer.

[4] Bruce J, Thornton AJ, Scott NW, Marfizo S, Powell R, Johnston M, Wells M, Heys SD, Thompson AM (2012). Chronic preoperative pain and psychological robustness predict acute postoperative pain outcomes after surgery for breast cancer. Br J Cancer.

[5] Burke AL, Mathias JL, Denson LA (2015). Psychological functioning of people living with chronic pain: a meta-analytic review. Br J Clin Psychol.

[6] Burns JW, Quartana PJ, Bruehl S (2008). Anger inhibition and pain: conceptualizations, evidence and new directions. J Behav Med.

[7] Callaghan BC, Xia R, Reynolds E, Banerjee M, Rothberg AE, Burant CF, Villegas-Umana E, Pop-Busui R, Feldman EL (2016). Association between metabolic syndrome components and polyneuropathy in an obese population. JAMA Neurol.

[8] Chou L, Brady SRE, Urquhart DM, Teichtahl AJ, Cicuttini FM, Pasco JA, Brennan-Olsen SL, Wluka AE (2016). The association between obesity and low back pain and disability is affected by mood disorders: a population-based, cross-sectional study of men. Medicine (Baltimore).

[9] Chou R, Gordon DB, de Leon-Casasola OA, Rosenberg JM, Bickler S, Brennan T, Carter T, Cassidy CL, Chittenden EH, Degenhardt E, Griffith S, Manworren R, McCarberg B, Montgomery R, Murphy J, Perkal MF, Suresh S, Sluka K, Strassels S, Thirlby R, Viscusi E, Walco GA, Warner L, Weisman SJ, Wu CL (2016). Management of postoperative pain: a clinical practice guideline from the american pain society, the american society of regional anesthesia and pain medicine, and the american society of anesthesiologists’ committee on regional anesthesia, executive committee, and administrative council. J Pain.

[10] Dableh LJ, Yashpal K, Henry JL (2011). Neuropathic pain as a process: reversal of chronification in an animal model. J Pain Res.

[11] Dimova V, Lötsch J, Hühne K, Winterpacht A, Heesen M, Parthum A, Weber PG, Carbon R, Griessinger N, Sittl R, Lautenbacher S (2015). Association of genetic and psychological factors with persistent pain after cosmetic thoracic surgery. J Pain Res.

[12] Dworkin RH, Turk DC, Farrar JT, Haythornthwaite JA, Jensen MP, Katz NP, Kerns RD, Stucki G, Allen RR, Bellamy N, Carr DB, Chandler J, Cowan P, Dionne R, Galer BS, Hertz S, Jadad AR, Kramer LD, Manning DC, Martin S, McCormick CG, McDermott MP, McGrath P, Quessy S, Rappaport BA, Robbins W, Robinson JP, Rothman M, Royal MA, Simon L, Stauffer JW, Stein W, Tollett J, Wernicke J, Witter J, IMMPACT (2005). Core outcome measures for chronic pain clinical trials: IMMPACT recommendations. Pain.

[13] Edwards RR, Dworkin RH, Sullivan MD, Turk DC, Wasan AD (2016). The Role of Psychosocial Processes in the Development and Maintenance of Chronic Pain. J Pain.

[14] Fayaz A, Croft P, Langford RM, Donaldson LJ, Jones GT (2016). Prevalence of chronic pain in the UK: a systematic review and meta-analysis of population studies. BMJ Open.

[15] Ferlay J, Soerjomataram I, Dikshit R, Eser S, Mathers C, Rebelo M, Parkin DM, Forman D, Bray F (2015). Cancer incidence and mortality worldwide: sources, methods and major patterns in GLOBOCAN 2012. Int J Cancer.

[16] Fillingim RB, Ohrbach R, Greenspan JD, Knott C, Diatchenko L, Dubner R, Bair E, Baraian C, Mack N, Slade GD, Maixner W (2013). Psychological factors associated with development of TMD: the OPPERA prospective cohort study. J Pain.

[17] Fitzmaurice C, Allen C, Barber RM, Barregard L, Bhutta ZA, Brenner H, Dicker DJ, Chimed-Orchir O, Dandona R, Dandona L, Fleming T, Forouzanfar MH, Hancock J, Hay RJ, Hunter-Merrill R, Huynh C, Hosgood HD, Johnson CO, Jonas JB, Khubchandani J, Kumar GA, Kutz M, Lan Q, Larson HJ, Liang X, Lim SS, Lopez AD, MacIntyre MF, Marczak L, Marquez N, Mokdad AH, Pinho C, Pourmalek F, Salomon JA, Sanabria JR, Sandar L, Sartorius B, Schwartz SM, Shackelford KA, Shibuya K, Stanaway J, Steiner C, Sun J, Takahashi K, Vollset SE, Vos T, Wagner JA, Wang H, Westerman R, Zeeb H, Zoeckler L, Abd-Allah F, Ahmed MB, Alabed S, Alam NK, Aldhahri SF, Alem G, Alemayohu MA, Ali R, Al-Raddadi R, Amare A, Amoako Y, Artaman A, Asayesh H, Atnafu N, Awasthi A, Saleem HB, Barac A, Bedi N, Bensenor I, Berhane A, Bernabe E, Betsu B, Binagwaho A, Boneya D, Campos-Nonato I, Castaneda-Orjuela C, Catala-Lopez F, Chiang P, Chibueze C, Chitheer A, Choi JY, Cowie B, Damtew S, das Neves J, Dey S, Dharmaratne S, Dhillon P, Ding E, Driscoll T, Ekwueme D, Endries AY, Farvid M, Farzadfar F, Fernandes J, Fischer F, TT GH, Gebru A, Gopalani S, Hailu A (2017). Global, regional, and national cancer incidence, mortality, years of life lost, years lived with disability, and disability-adjusted life-years for 32 cancer groups, 1990 to 2015: a systematic analysis for the global burden of disease study. JAMA Oncol.

[18] Good PI (2006). Resampling methods: a practical guide to data analysis.

[19] Guyon I, Andr E (2003). An introduction to variable and feature selection. J Mach Learn Res.

[20] Hussain SM, Urquhart DM, Wang Y, Dunstan D, Shaw JE, Magliano DJ, Wluka AE, Cicuttini FM (2016). Associations between television viewing and physical activity and low back pain in community-based adults: a cohort study. Medicine (Baltimore).

[21] Janssen SA (2002). Negative affect and sensitization to pain. Scand J Psychol.

[22] Julius D, Basbaum AI (2001). Molecular mechanisms of nociception. Nature.

[23] Juran JM (1975). The non-Pareto principle; Mea culpa. Qual Prog.

[24] Kaunisto MA, Jokela R, Tallgren M, Kambur O, Tikkanen E, Tasmuth T, Sipilä R, Palotie A, Estlander A-M, Leidenius M, Ripatti S, Kalso EA (2013). Pain in 1,000 women treated for breast cancer: a prospective study of pain sensitivity and postoperative pain. Anesthesiology.

[25] Kehlet H, Jensen TS, Woolf CJ (2006). Persistent postsurgical pain: risk factors and prevention. Lancet.

[26] Kendall MG (1938). A new measure of rank correlation. Biometrika.

[27] Knaster P, Estlander AM, Karlsson H, Kaprio J, Kalso E (2016). Diagnosing depression in chronic pain patients: DSM-IV major depressive disorder vs. beck depression inventory (BDI). PLoS ONE.

[28] Kullback S, Leibler RA (1951) On Information and Sufficiency.pp 79–86. 10.1214/aoms/1177729694

[29] Lasselin J, Kemani MK, Kanstrup M, Olsson GL, Axelsson J, Andreasson A, Lekander M, Wicksell RK (2016). Low-grade inflammation may moderate the effect of behavioral treatment for chronic pain in adults. J Behav Med.

[30] Lautenbacher S, Huber C, Schofer D, Kunz M, Parthum A, Weber PG, Roman C, Griessinger N, Sittl R (2010). Attentional and emotional mechanisms related to pain as predictors of chronic postoperative pain: a comparison with other psychological and physiological predictors. Pain.

[31] Linton SJ, Nicholas MK, MacDonald S, Boersma K, Bergbom S, Maher C, Refshauge K (2011). The role of depression and catastrophizing in musculoskeletal pain. Eur J Pain.

[32] Lötsch J, Ultsch A, Kalso E (2017) Prediction of persistent post-surgery pain by preoperative cold pain sensitivity: Biomarker development with machine-learning-derived analysis. Br J Anaesth aex236 10.1093/bja/aex236 29121286

[33] Margari F, Lorusso M, Matera E, Pastore A, Zagaria G, Bruno F, Puntillo F, Margari L (2014). Aggression, impulsivity, and suicide risk in benign chronic pain patients - a cross-sectional study. Neuropsych Dis Trea.

[34] McCracken LM, Gross RT, Aikens J, Carnrike CL (1996). The assessment of anxiety and fear in persons with chronic pain: a comparison of instruments. Behav Res Ther.

[35] McGrayne SB (2011). The Theory That Would Not Die: How Bayes’ Rule Cracked the Enigma Code, Hunted Down Russian Submarines & Emerged Triumphant from Two Centuries of Controversy.

[36] Mejdahl MK, Andersen KG, Gartner R, Kroman N, Kehlet H (2013). Persistent pain and sensory disturbances after treatment for breast cancer: six year nationwide follow-up study. Bmj.

[37] Meretoja TJ, Leidenius MH, Tasmuth T, Sipila R, Kalso E (2014). Pain at 12 months after surgery for breast cancer. JAMA.

[38] Motaghedi R, Bae JJ, Memtsoudis SG, Kim DH, Beathe JC, Paroli L, YaDeau JT, Gordon MA, Maalouf DB, Lin Y, Ma Y, Cunningham-Rundles S, Liu SS (2014). Association of obesity with inflammation and pain after total hip arthroplasty. Clin Orthop Relat Res.

[39] Murphy KP (2012). Machine Learning: A Probabilistic Perspective.

[40] Okifuji A, Hare BD (2015). The association between chronic pain and obesity. J Pain Res.

[41] Paice JA, Portenoy R, Lacchetti C, Campbell T, Cheville A, Citron M, Constine LS, Cooper A, Glare P, Keefe F, Koyyalagunta L, Levy M, Miaskowski C, Otis-Green S, Sloan P, Bruera E (2016). Management of chronic pain in survivors of adult cancers: american society of clinical oncology clinical practice guideline. J Clin Oncol.

[42] Pareto V (1909). Manuale di economia politica, Milan: Società editrice libraria, revised and translated into French as Manuel d’économie politique.

[43] Pergolizzi JV, Gharibo C, Ho K-Y (2015). Treatment considerations for cancer pain: a global perspective. Pain Pract.

[44] Portenoy RK (1992). Cancer pain: pathophysiology and syndromes. Lancet.

[45] R Development Core Team (2008) R: a language and environment for statistical computing

[46] Ray L, Lipton RB, Zimmerman ME, Katz MJ, Derby CA (2011). Mechanisms of association between obesity and chronic pain in the elderly. Pain.

[47] Schaible HG (2007) Peripheral and central mechanisms of pain generation. Handb Exp Pharmacol:3–28 10.1007/978-3-540-33823-9_1 17087118

[48] Schou Bredal I, Smeby NA, Ottesen S, Warncke T, Schlichting E (2014). Chronic pain in breast cancer survivors: comparison of psychosocial, surgical, and medical characteristics between survivors with and without pain. J Pain Symptom Manage.

[49] Sipilä R, Estlander A-M, Tasmuth T, Kataja M, Kalso E (2012). Development of a screening instrument for risk factors of persistent pain after breast cancer surgery. Br J Cancer.

[50] Spielberger CD (1999) Staxi-2: state-trait anger expression inventory-2; professional manual. PAR, Psychological Assessment Resources

[51] Spielberger CD, Gorsuch RL, Lushene RE (1970). The State-Trait Anxiety Inventory (test manual).

[52] Tasmuth T, von Smitten K, Kalso E (1996). Pain and other symptoms during the first year after radical and conservative surgery for breast cancer. Br J Cancer.

[53] Torrance N, Smith BH, Bennett MI, Lee AJ (2006). The epidemiology of chronic pain of predominantly neuropathic origin. Results from a general population survey. J Pain.

[54] Trimble EL, Ungerleider RS, Abrams JA, Kaplan RS, Feigal EG, Smith MA, Carter CL, Friedman MA (1993). Neoadjuvant therapy in cancer treatment. Cancer.

[55] Udovičić M, Baždarić K, Bilić-Zulle L, Petrovečki M (2007). What we need to know when calculating the coefficient of correlation?. Biochem Med.

[56] Ultsch A, Lötsch J (2015). Computed ABC Analysis for rational selection of most informative variables in multivariate data. PLoS ONE.

[57] Vlaeyen JW, Crombez G, Linton SJ (2016). The fear-avoidance model of pain. Pain.

[58] Vlaeyen JW, Linton SJ (2012). Fear-avoidance model of chronic musculoskeletal pain: 12 years on. Pain.

[59] Weiss SM, Indurkhya N (1995). Rule-based machine learning methods for functional prediction. J Artif Int Res.

[60] Wright LJ, Schur E, Noonan C, Ahumada S, Buchwald D, Afari N (2010). Chronic pain, overweight, and obesity: findings from a community-based twin registry. J Pain.

