# Supplementary material for: Machine-learning-derived classifier predicts absence of persistent pain after breast cancer surgery with high accuracy
Source: Breast Cancer Res Treat. 2018 Jun 6;171(2):399–411. doi: 10.1007/s10549-018-4841-8 (PMC6096884; doi:10.1007/s10549-018-4841-8)
Supplement: Supplementary file 1 — Supplementary material 1 (DOCX 43 KB) [file 10549_2018_4841_MOESM1_ESM.docx]

# Detailed description of the data analysis

The application of the previously-proposed six-factor risk index for persisting pain was performed according to the instructions in Table A1 in the appendix of [17]. In brief, the six items “age”, “chronic pain of any kind”, “number of previous operations”, “body mass index”, “preoperative pain in the area to be operated on” and “smoking” were weighted according to these instructions. For example, age ≤ 39 years was given a weight of 0, age 40 – 69 years a weight of 8, and age ≥ 70 years a weight of 16. The respective weights for the six parameters were added and a sum ≥ 20 was taken as predicting persistent pain.

In addition, a novel and independently-designed analysis was performed to assess whether the classification performance could be improved from that obtained with the previously proposed diagnostic tool. Data analysis was performed using the MATLAB (MathWorks, Natick, MS, USA) and R (version 3.2.3 for Linux; <http://CRAN.R-project.org/> [16]) software packages. We used supervised machine-learning [14] and feature-selection techniques [8] to identify parameters from the data acquired before surgery and up to the sixth month after surgery that could predict the presence or absence of persisting pain in the area operated on during the three-year follow-up available for the present analysis. In this approach, the goal is to learn a mapping from inputs *x* to outputs *y*, given a labelled set of input-output pairs $D=\left\{ \left( x_{i},y_{i} \right) | x_{i} X, y_{i}Y, i=1\ldots n \right\}$. Here, *D* denotes the so-called “data space^[[1]](#footnote-2)^” with a predefined division into an input space *X* comprising *x_i_* as the features (parameters) possibly predicting the diagnosis, and the output space *Y* comprising *y_i_* as the classes or diagnostic groups.

The analysis was performed in four main steps (Figure 2 in the main document). **First**, the so-called “output space” was established by identifying the groups or “classes” of patients with respect to persistent pain. In the following, the term “persisting pain group” and “non-persisting pain group” or “classes” will be used when referring to these groups. Subsequently, the input or feature space was analyzed to identify those parameters that provided a valid assignment of a patient to the classes of persisting or non-persisting pain. This included the **second** analytical step comprising a “feature” pre-selection, during which all parameters were analyzed with respect to differences between the pain-persistence groups established in the output space. Those parameters that had passed that step were taken into the **third** analytical step of feature selection, which eliminated all parameters distinguishing between pain persistence groups but not providing additional relevant information justifying their inclusion in a tool (“classifier”) predictive of persisting pain. The remaining parameters, in the following called “features”, were taken into the **fourth** step of the data analysis in which the predictive tool or “classifier” was constructed and assessed with respect to standard test performance measures. Details of this machine-learned data analysis are described below.

### Class identification for persisting pain

The first analytical step addressed the output space (for definition, see previous section), i.e., the identification of the groups or classes of patients with respect to persisting pain. To obtain the output space for machine-learning, a robust classification of the patients into women who develop persisting pain versus those who clearly do not was used (Figure 2 in the main document, left part). This classification was derived from the NRS ratings of pain intensity in the area of surgery at 12, 24, and 36 months. A value of NRS = 3 was used as a clinically-established cut-off for no or mild pain (NRS 0 - 3) versus NRS = 4 – 10 for moderate to severe pain despite analgesic medication [6, 9]. Using these criteria, patients with NRS ≤ 3 at month 36 after surgery $({NRS}_{month36}\leq3)$ were identified as belonging to the “non-persisting pain” group, while those with $({NRS}_{month36}\geq4)$ had “persisting pain”. A further criterion for the “non-persisting pain” group was the presence of no to mild pain (NRS ≤ 3) at months 12, 24 and 36 after surgery, i.e., ${NRS}_{month12..month36}\leq3$. In order to enhance the contrast between the groups, the “persisting pain” group was further characterized by having rated pain as NRS > 0 at all time points of 12, 24 and 36 months after surgery and with no tendency toward amelioration of pain, i.e. no decrease in NRS ratings. Hence, the “persisting pain” group was observed under the following conditions: ${NRS}_{month36}\geq4 and {NRS}_{month12..month36}>0 and ({NRS}_{month36}-{NRS}_{month24})\geq0$. This enabled the classification of 833 patients. The classification of the remaining 167 patients was achieved by applying the following rules: If all available ratings were ${NRS}_{month12..month36}\geq4$ then the case was subsumed to the “persisting pain” group (n = 21). By contrast, if the available ratings were ${NRS}_{month6..month36}\leq3$ and $({NRS}_{month24}<{NRS}_{month6})$, i.e. a decrease over time, then the case was subsumed into the “non-persisting pain” group (n = 146). This finally resulted in a binary classification which was then approached by means of machine-learning.

### Feature pre-selection

Following the above-described establishment of the “output space” consisting of a robust group structure of clinical interest (persisting pain or non-persisting pain), “feature” selection was applied to the “input space” (Figure 2 in the main document, right part) of the 542 parameters, comprising 52 continuous-scaled, 154 discrete and 336 binary variables, that had been acquired preoperatively and during the six months after surgery. This step additionally included the combination (aggregation) of pain-related parameters evaluated at different time points. Supervised “greedy” machine-learning was applied to identify “features” that best distinguished between the persisting and non-persisting pain groups. Secondly, from the candidates (original or aggregated “features”) identified during the first step, “features” that provided the largest contributions to the sensitivity and specificity of the clinical diagnostic tool were allocated to the final classifier. “Feature” pre-selection started with separate analyses of available parameters (continuous/interval scaled, discrete or binary variables), with respect to differences of their distributions between the persisting or non-persisting pain groups. During this process, correlations between variables were not analyzed, except for the closely-related parameters “body weight“ and “BMI,” of which the slightly better performing variable BMI was selected. For each variable type, appropriate statistical or informatics criteria were applied to analyze whether they displayed class differences according to standard approaches in machine learning [5, 18, 20]. This will be described in the following paragraphs.

#### Feature pre-selection of interval-scaled variables

The 52 variables available as continuous (interval-scaled) vectors $X\in\mathcal{R}^{d}$ (feature vector) were mapped to the discrete classes $Y\in Y$ with classes *y_1_* and *y_2_* accounting for the inclusion in or exclusion from the “persisting pain” group of a patient (see above). The classification rule ${\psi: \mathcal{R}}^{d}\to Y$ assigned a class label (persisting or non-persisting pain) to the data on the basis of the observation of its feature vector by partitioning the feature space in two decision regions *D_1_*, *D_2_* (yes or no, respectively) such that $D_{i}=\left\{ x\in\mathcal{R}^{d} | \psi\left( x \right)=y_{i} \right\}$. The border between the two classes was obtained by applying the Bayesian decision rule as $\psi_{B}\left( x \right)=if p_{Y|X}\left( y_{1} | x \right)>p_{Y|X}\left( y_{2} | x \right) then y_{1}else y_{2}$ [12]. For 17 continuous variables, a decision border, i.e. the point where the decision rule $\psi_{B}\left( x \right)$changes its value, could be obtained. For the other continuous variables, the distributions among patients belonging to a class and those not belonging to that class were virtually identical, not allowing a separation of the persisting pain and non-persisting pain groups.

#### Feature pre-selection of discrete variables

Of the 154 variables available as discrete vectors $X\in N^{d}$ (feature vector), 41 were not directly related to pain while 113 addressed pain or its impact on the patient’s life. Specifically, pain-related parameters included questions about how much the pain has affected the patient’s life or disturbed the patient’s sleep: these were asked separately for relevant body locations including the operated breast, the axilla, the upper arm, the lower arm, hand/fingers and the associated joints of the same side, and any other body location (“elsewhere”). In the machine-learning approach aimed at identifying the most informative parameters (features) for patient classification, a major focus on pain-related parameters could have led to the selection of many closely-related parameters, disregarding other clinical parameters (features). Therefore, an additional aggregation step was implemented combining several pain-related parameters as shown in Table 1 and described in detail in the following paragraph.

Aggregation for body area was obtained as the arithmetic mean of the ratings of pain-related impact on the patient’s life asked separately for several anatomical regions, which are specified in full detail in Table 1. For example, the average degree to which pain in the upper extremity had influenced the patient’s life at month 1 after surgery was obtained as ${AffectingLife}_{extremity,month1}=\frac{1}{5}\cdot\sum\begin{aligned} {AffectingLife}_{axilla,month1},{AffectingLife}_{upper arm, month1}, \\ {AffectingLife}_{elbow, month1},{AffectingLife}_{lower arm, month1}, \\ {AffectingLife}_{hand,month1} \end{aligned}$. Aggregation for time was obtained as the arithmetic mean of the ratings of the pain-related impact on the patient’s life asked for the same anatomical region at several different time points after surgery. For example, the average degree to which pain in the axilla had affected the patient’s life was obtained as the mean of the values obtained at month 1 and month 2 after surgery. In the same manner, averages were calculated for pain in the upper arm, the lower arm, the hand, and the joints and fingers, that influenced the patient’s life. These provided the aggregated variables “*AffectingLife_UpperArmMean_*”, “*AffectingLife_LowerArmMean_*”, “*AffectingLife_HandMean_*” and “*AffectingLife_JointsMean_*”, respectively. Similarly, the average degree to which pain in the axilla had disturbed the patient’s sleep was obtained as the mean ratings of sleep disturbances during month 1 and month 6 after surgery, and further averages were calculated for the pain in the operated breast and the joints, which were inquired about for sleep effects, providing the aggregated variables “*SleepDisturbance_PainBreast_*” and “*SleepDisturbance_PainJoints_*”, respectively. At one and six months after surgery, patients had been asked to rate the pain during the past week in the breast, the axilla, the upper arm, lower arm, hand or in some other location, which when averaged provided the aggregated variables “*MeanPainPastWeek_Breast_*“, “*MeanPainPastWeek_Axilla_*”, “*MeanPainPastWeek_UpperArm_*”, “*MeanPainPastWeek_LowerArm_*”, “*MeanPainPastWeek_Hand_*” and “*MeanPainPastWeek_somewhere_*”, respectively.

Subsequently, the deviation between the parameter distributions in the n = 779 “non-persisting pain” group (P) and the n = 70 “persisting pain” group (Q) was analyzed. This difference in distributions was expressed using the symmetrized Kullback–Leibler divergence [11] $D_{SKL}\left( P||Q \right)=D_{KL}\left( P||Q \right)+ D_{KL}\left( Q||P \right)$ with $D_{KL}\left( P||Q \right)=\sum_{i} p_{i}\cdot log\frac{P_{i}}{q_{i}}$. Pain ratings acquired for particular locations were averaged over body locations or over different time points (for details, see Table 1 of the main document). This resulted in 21 possible discrete parameters (features), which passed this feature pre-selection step. Regarding the pain ratings, we asked patients at different time points to rate the pain intensity in different locations (e.g. axilla, upper arm, lower arm etc.; for details, see Table 1 of the main document and the Supplementary Table), using different measures (such as how much the pain affected daily activities or the mean pain intensity during the previous week). The means of the respective measurements at the different time points were calculated for each body location.

#### Feature pre-selection of binary variables

The 336 variables available as binary (“yes”/”no”) vectors $X\in B^{d}$ (feature vector) were mapped to the discrete classes $Y\in Y$ using the odds ratio combined with prediction accuracy. Specifically, the odds ratio (*OR*) related the probability, *A*, of belonging to the “persisting pain” group in the presence of a feature to the probability, *B*, of belonging to that group in the absence of the same feature, i.e. $OR=\frac{P\left( A \right)\cdot\left( 1-P\left( B \right) \right)}{P\left( B \right)\cdot\left( 1-P\left( A \right) \right)}$. None of the binary variables achieved a better accuracy than obtained by chance. Therefore, the binary variables were excluded as effective predictive parameters (features).

### Feature (parameter) selection

The third analytical step (Figure 2 in the main document, right part) targeted the selection of those parameters that qualified for inclusion in a predictive tool (classifier) of the pain persistence groups (output space). During this step, those parameters were eliminated that had passed feature (parameter) pre-selection but failed to provide sufficient relevant information justifying their inclusion in the predictive tool. Thus, following the pre-selection of 38 candidate parameters (features) for the classifier for persistent pain after breast cancer surgery (17 continuous and 21 discrete variables), those parameters were selected that provided significant contributions to the classification accuracy while those parameters that lacked such significant contribution were eliminated. Therefore, in this analysis, taking only the most informative parameters, some parameters were excluded, despite their displaying significant differences between pain persistence groups, if they did not provide further improvement of group classification than already obtained with other parameters. Specifically, the aim of the analysis was to identify a parameter set that provided the best classification of patients, as opposed to merely analyzing each parameter with respect to group differences. The selection of the most suitable parameters for the final classifier was achieved by applying a calculated ABC analysis [19]. This is an inventory categorisation technique originally developed for problems in economics to search for a subset with minimum effort that gives the maximum yield [10, 15]. It divides a set of positive (yield) data into three disjoint subsets called “A”, “B” and “C”. Subset “A” comprised "the important few" which promise maximum gain with low effort whereas subset “C” comprises "the trivial many", i.e., non-profitable values. Subset “B” comprises the values where effort and gain are in balance. Its results are visualized in an ABC curve [7] representing the distribution function of effort versus cumulative yield.

To obtain a robust selection of parameters (features), the process was repeated using Monte Carlo resampling. Specifically, for the 17 continuous parameters the best possible Bayesian decision limits were identified iteratively. For the discrete parameters, all possible decision thresholds were used. Subsequently, a preliminary classification obtained with all parameters was analyzed to test specificity versus sensitivity. The contribution of each candidate factor to the area under the specificity versus sensitivity plot (area under the curve, AUC) [13] was subjected to ABC analysis, which identified the parameters belonging to set “A”, i.e., those features that promise the most gain in terms of correct clinical diagnosis. During resampling, this procedure was repeated 1,000 times with 50% of the patients randomly (group-wise) drawn from the complete data set, i.e., a class-size proportional subsample of 50 % of the cases was randomly chosen and the feature selection was performed using this data subset. Only parameters that always belonged to ABC set “A” (mean and confidence interval of the placement from the set “A” threshold) were selected for further consideration as components of the desired classifier. Six continuous and 15 discrete parameters passed this feature selection step.

### Establishment of a predictive classifier

#### Creation of the classifier

In the forth analytical step (Figure 2 in the main document, bottom right), from the 21 single or aggregated variables (Table 1 in the main document; 6 continuous, 15 discrete variables; see also Figure 2 in the main document) that had passed the two-step process of feature selection and therefore qualified as candidate predictors of persisting pain group membership, a diagnostic instrument consisting of a rule-based classifier [21] was built, forming a questionnaire with “yes/no” items (decision rules) and used the decision borders established as described above, i.e., Bayesian limits for continuous data, Kullback–Leibler divergence-based for ordinal data and odd ratio-based for binary data.

Specifically, applying a “brute force” approach, all possible combinations of the identified rules were tried, with 1 to 21 rules. For every combination, the product of sensitivity and specificity of the obtained prediction was calculated. That is, the test performance parameters were calculated if any one of the 21 rules applied, any two of the 21 rules applied and so on. The sensitivity and specificity of the test for assignment to a group were calculated using standard equations [2], i.e., *sensitivity [%] = 100 · true positives / (true positives + false negatives)* and *specificity [%] = 100 · true negatives / (true negatives + false positives)* [2]. This identified specific “yes/no” thresholds for each of the 21 parameters (single or aggregated variables) and an optimum threshold for patient classification. Hence, the classifier consisted of a set of questions from which the sum score of the positive responses served as the diagnostic tool.

#### Validation of the classifier

The classifier was subsequently analyzed for test performance. In addition, to test sensitivity (calculated as described above), the negative predictive value, indicating how unlikely it was that the patient would develop persisting pain when the test was negative, was calculated as *NPV [%] = 100 · true negative / (true negative + false negative)* [1], the test accuracy was obtained as *accuracy [%] = number of correct diagnoses / total number of diagnoses* [13], and the balanced test accuracy was obtained as *balanced* *accuracy [%] = (sensitivity + specificity) / 2* [3].

The generalizability of the classifier was assessed using (i) a cross-validation approach in the learning data subset, i.e. the data set comprising the n = 853 women included in classifier construction, and (ii) by applying it on the n = 147 patients with complete data who were excluded from the model building because of not satisfying the strict criteria of persisting versus non-persisting pain. In the learning data subset, the classifier’s performance was tested in a Monte Carlo approach [4] running 1,000 times on a randomly-picked 50% of the total sample. Specifically, (i) in each run, a class-size proportional subsample of 50 % of the cases was randomly chosen as a new training data subset. The rules were reestablished on this sample in the same manner as described above and the classifier performance was tested on the remaining 50 % of the sample that served as the test data subset. In the patients excluded from classifier establishment (ii), the classifier’s performance was tested by classifying these patients only according to the first criterion of the NRS-based classification, i.e., ${NRS}_{month36}\leq3$ versus ${NRS}_{month36}\geq4$.

# References

1. Altman DG, Bland JM (1994) Diagnostic tests 2: Predictive values. BMJ 309:102.

2. Altman DG, Bland JM (1994 ) Diagnostic tests. 1: Sensitivity and specificity. Br. Med. J. 308:1552.

3. Brodersen KH, Ong CS, Stephan KE, Buhmann JM (2010) The Balanced Accuracy and Its Posterior Distribution. In: Pattern Recognition (ICPR), 2010 20th International Conference on. p 3121-3124

4. Caflisch RE (1998) Monte Carlo and quasi-Monte Carlo methods. Acta Numerica 7:1-49. doi: doi:10.1017/S0962492900002804

5. Caldas J, Gehlenborg N, Faisal A, Brazma A, Kaski S (2009) Probabilistic retrieval and visualization of biologically relevant microarray experiments. BMC Bioinformatics 10:P1. doi: 10.1186/1471-2105-10-s13-p1

6. Dworkin RH, Turk DC, Farrar JT, Haythornthwaite JA, Jensen MP, Katz NP, Kerns RD, Stucki G, Allen RR, Bellamy N, Carr DB, Chandler J, Cowan P, Dionne R, Galer BS, Hertz S, Jadad AR, Kramer LD, Manning DC, Martin S, McCormick CG, McDermott MP, McGrath P, Quessy S, Rappaport BA, Robbins W, Robinson JP, Rothman M, Royal MA, Simon L, Stauffer JW, Stein W, Tollett J, Wernicke J, Witter J, IMMPACT (2005) Core outcome measures for chronic pain clinical trials: IMMPACT recommendations. Pain 113:9-19. doi: 10.1016/j.pain.2004.09.012

7. Gastwirth JL, Glauberman M (1976) The Interpolation of the Lorenz curve and Gini index from grouped data. Econometrica 44:479-483.

8. Guyon I, Andr, #233, Elisseeff (2003) An introduction to variable and feature selection. J. Mach. Learn. Res. 3:1157-1182.

9. Johansen A, Romundstad L, Nielsen CS, Schirmer H, Stubhaug A (2012) Persistent postsurgical pain in a general population: prevalence and predictors in the Tromso study. Pain 153:1390-1396. doi: 10.1016/j.pain.2012.02.018

10. Juran JM (1975) The non-Pareto principle; Mea culpa. Quality Progress 8:8-9.

11. Kullback S, Leibler RA (1951) On Information and Sufficiency.79-86. doi: 10.1214/aoms/1177729694

12. McGrayne SB (2011) The Theory That Would Not Die: How Bayes' Rule Cracked the Enigma Code, Hunted Down Russian Submarines & Emerged Triumphant from Two Centuries of Controversy. Yale University Press, New Haven, CT, USA

13. Metz CE (1978) Basic principles of ROC analysis. Semin Nucl Med 8:283-298.

14. Murphy KP (2012) Machine Learning: A Probabilistic Perspective. The MIT Press

15. Pareto V (1909) Manuale di economia politica, Milan: Società editrice libraria, revised and translated into French as Manuel d’économie politique. In:Giard et Briére, Paris

16. R Development Core Team (2008) R: A Language and Environment for Statistical Computing.

17. Sipilä R, Estlander A-M, Tasmuth T, Kataja M, Kalso E (2012) Development of a screening instrument for risk factors of persistent pain after breast cancer surgery. Br J Cancer 107:1459-1466. doi: 10.1038/bjc.2012.445

18. Tripathi A, Klami A, Orešič M, Kaski S (2011) Matching samples of multiple views. Data Mining and Knowledge Discovery 23:300-321. doi: 10.1007/s10618-010-0205-7

19. Ultsch A, Lötsch J (2015) Computed ABC Analysis for Rational Selection of Most Informative Variables in Multivariate Data. PLoS One 10:e0129767. doi: 10.1371/journal.pone.0129767

20. Venna J, Kaski S (2007) Nonlinear dimensionality reduction as information retrieval. In: Artificial Intelligence and Statistics. p 572-579

21. Weiss SM, Indurkhya N (1995) Rule-based machine learning methods for functional prediction. J. Artif. Int. Res. 3:383-403.

1. **Data space** is the space in which the n-dimensional feature vectors can be observed. A particular dimension’s data space may be bound to certain ranges. For example, [0…100]^n^ may be a data space of percentages. Sometimes data space is used synonymously with feature space.

   **Feature vector** is a d-dimensional vector of numerical features (parameters) that represent one data object (case). The complete set of features in a data set forms the so-called “**feature space**.”

   The **output space** *Y* consists of possible classes, in a medical context, diagnoses, y_i,_ presently defined as persistent or non-persistent postoperative pain. [↑](#footnote-ref-2)
